# Effect of phosphorus fraction in shaping bacterial and archaeal community succession in the largest hydrologically connected lake of Northeast Asia

**DOI:** 10.3389/fmicb.2026.1844785

**Published:** 2026-07-28

**Authors:** Zijian Xie, Xinghong Liu, Bo Bo, Weiwei Wei, Chunhua Li, Chun Ye

**Affiliations:** 1 Key Laboratory of Drinking Water Source Protection of the Ministry of Ecology and Environment, National Engineering Laboratory for Lake Pollution Control and Ecological Restoration, Chinese Research Academy of Environmental Sciences, Beijing, China; 2 College of Environmental and Safety Engineering, Shenyang University of Chemical Technology, Shenyang, China

**Keywords:** beta diversity, hydrologically connected lake, microbial ecological indicators, phosphorus fractions, Xingkai Lake

## Abstract

Microbial beta diversity and its components are key ecological indicators for understanding community assembly in lake sediments, yet their coupling with phosphorus (P) fractions remains poorly understood in hydrologically connected lake systems. In this study, sediment cores were collected from Xingkai Lake, the largest freshwater lake in Northeast Asia with a unique twin lake structure, and were analyzed using metagenomic sequencing combined with sequential P fractionation. Results showed that total beta diversity and species turnover for bacteria and archaea increased significantly with sediment depth in both lakes, with faster turnover rates in Daxingkai Lake. Nestedness was generally not significant in Daxingkai Lake but showed a significant positive trend with depth for archaea in Xiaoxingkai Lake. The dominant P fraction in the Daxingkai Lake sediments were HCl-Pi and residual P, while NaOH-Pi dominated in Xiaoxingkai lake sediments. Organic P explained the largest proportion of bacterial beta diversity variation in Daxingkai Lake, while inorganic P was the primary driver in Xiaoxingkai Lake. Conversely, inorganic P dominated the archaeal beta diversity variation in Daxingkai, whereas organic P dominated in Xiaoxingkai. These findings demonstrate that species turnover is the dominant component of beta diversity along the sediment depth gradient. The contrasting roles of organic P and inorganic P in shaping microbial beta diversity highlight the importance of P resource partitioning in driving microbial community succession and provide a basis for developing microbial beta diversity indicators to support eutrophication assessment and sediment management in hydrologically connected lake systems.

## 1 Introduction

Microbial diversity is fundamental to maintaining the functional integrity and stability of lake aquatic ecosystems, which plays a crucial role in the decomposition of organic matter and the biogeochemical cycles of nutrients such as nitrogen (N) and phosphorus (P) (Zhang et al., 2018; Liu et al., 2023; Peng et al., 2024). For instance, phosphate-solubilizing and phosphate-accumulating bacteria directly regulate the release and sequestration of P in lake sediments (Song et al., 2024; Xie et al., 2025). In addition, microorganisms drive energy and matter transfer to higher trophic levels via protozoa and zooplankton grazing (Yuan et al., 2023; Foulquier et al., 2024). Microorganisms also sustain water body self-purification through adsorption, intracellular enrichment, redox reactions and methylation (Yuan et al., 2023; Song et al., 2024). Given their high sensitivity to environmental change, microbial community structure and functional genes serve as bioindicators of aquatic ecosystems health and stress (Michán et al., 2021; Song et al., 2024). More importantly, lake sediments serve as natural archives of environmental change, with different strata preserving a continuous record of historical environmental transformations in the lake and its catchment (Teixeira et al., 2021). The microbial community structure and functional genes preserved within these sediments reflect the dominant ecological processes and environmental conditions at the time of deposition (Teixeira et al., 2021; Song et al., 2024). Therefore, analyzing vertical microbial diversity patterns along sediment profiles allows comprehensive reconstruction of long-term environmental pressures, and is crucial for understanding lake ecosystems evolution and functional mechanisms.

Beta diversity is a key indicator for quantifying species composition differences among communities across different habitats (Van der Plas et al., 2023; Yuan et al., 2023). Beta diversity is often divided into two components, species turnover and nestedness (Gaytán et al., 2025). Species turnover, which reflects the complete replacement of species between sites, is the primary contributor to high beta diversity (Xu et al., 2022; Gaytán et al., 2025). Its ecological basis typically points to strong turnover is often associated with niche-based processes, where environmental heterogeneity or competitive interactions influence species distribution (Xu et al., 2022; Gaytán et al., 2025). For example, oxygen gradients in lake sediments can lead to different microbial communities, with aerobic bacteria dominating surface layers and anaerobic bacteria and archaea dominating deeper layers (Zhang et al., 2025). This community shift along the dissolved oxygen gradient is reflected in high turnover (Chen et al., 2023; Gaytán et al., 2025). Moreover, turnover tends to be the dominant component of beta diversity in systems with strong environmental filtering (Chen et al., 2023; Gaytán et al., 2025). In contrast, nestedness describes a non-random pattern of species loss, where the species assemblage at one site is a subset of a richer assemblage at another site (Ren et al., 2022; Xu et al., 2023). High nestedness is typically associated with non-selective disturbance or intense environmental filtering, retaining only species with higher environmental tolerance or broader niches (Ren et al., 2022; Xu et al., 2023).

Generally, microbial diversity declines from the active surface layer to the deep layer sediments (Qian et al., 2023; Gao H. et al., 2025). The deep community may constitute a depauperate subset of the surface community, a pattern that can generate a significant nestedness component (Ruhl et al., 2008; Qian et al., 2023; Gao Y. et al., 2025). Changes in these two beta diversity components, along with shifts in co-occurrence network structure, have been demonstrated to serve as early-warning signals for ecosystem state transitions (Schweiger and Laliberté, 2022; Gao H. et al., 2025). As environmental stress approaches an ecological threshold, systematic changes in microbial beta diversity patterns and species interaction networks often precede detectable changes in macroscopic ecosystem functions (Schweiger and Laliberté, 2022; Gao H. et al., 2025; Walling et al., 2025). Notably, the dominant succession process (turnover or nestedness) varies with ecosystem type and stressor characteristics. For instance, high beta diversity in the macrobenthic community of the Yellow River Delta intertidal zone is dominated by species turnover, driven by high environmental heterogeneity and frequent physical disturbance (Wang et al., 2024; Fu et al., 2025). Conversely, Lauraceae plant communities in the Qinghai-Tibet Plateau and forest ecotones show high nestedness as the dominant pattern, attributed to species-selective extinction and distribution constraints from historical and contemporary climatic heterogeneity (Li et al., 2021). Therefore, analyzing bacterial and archaeal beta diversity and disentangling turnover and nestedness contributions across sediment depths and different trophic lakes provides an effective approach to understand microbial community succession and response to environmental stress.

P is a limiting nutrient in lake ecosystems, serving as a key driver of eutrophication and algal blooms (Woolway et al., 2024; Li et al., 2025). With external P inputs effectively controlled, internal P release from sediments has become the critical bottleneck for lake ecological restoration (Woolway et al., 2024; Li et al., 2025). Lake sediments act as a storage reservoir for P in aquatic systems, with storage capacities hundreds to thousands of times greater than that of the overlying water (Yu et al., 2023; Li et al., 2025). Under eutrophic conditions, sediments are not merely passive sinks for P but can become significant internal pollution sources under specific environmental conditions (Agstam-Norlin et al., 2020; Woolway et al., 2024). Sedimentary P occurs in multiple chemical forms, and different fractions exhibit distinct bioavailability and environmental behaviors (Psenner, 1988; Yuan et al., 2023). Additionally, microorganisms play key roles in the transformation of sedimentary P forms, playing indispensable roles in the dissolution, mineralization, immobilization, and conversion of P (Yuan et al., 2023; Xie et al., 2025). In turn, microbial community structure and function are profoundly influenced by the forms, concentration and bioavailability of P (Yuan et al., 2023; Xie et al., 2025). Notably, NaOH-Pi has been identified as a major factor associated with bacteria and archaea beta diversity in lakes, and phosphate availability fundamentally shapes the community structure of bacteria and archaea in aquatic ecosystems (Yuan et al., 2023; Woolway et al., 2024). Therefore, investigating the interactions between P fractions and bacterial/archaeal community succession in eutrophic lake sediments is essential for understanding the microbial mechanisms driving internal P release.

Xingkai Lake, the largest freshwater lake in Northeast Asia and an important cross-border lake, has been facing a continuous decline in water quality and increasingly prominent eutrophication (Li et al., 2025). P is the key limiting nutrient responsible for the degradation of water environment in Xingkai Lake (Li et al., 2025). Sediment P release is the core process regulating lake eutrophication (Xie et al., 2023; Li et al., 2025). Notably, Xingkai Lake features a unique twin-lake structure comprising of Daxingkai Lake and Xiaoxingkai Lake, which differ markedly in morphology, hydrology and ecological function (Xie et al., 2023; Li et al., 2025). Two lakes are hydrologically connected through a sluice gate. This provides a natural comparative setting to investigate the differences in sedimentary P fractions and their coupling to microbial community assembly between the two lakes, while also enabling the exploration of their intrinsic hydrological links (Xie et al., 2023; Li et al., 2025). Despite growing recognition that microbial beta diversity serves as a potential ecological indicator, few studies have systematically linked its turnover and nestedness components to specific P fractions along sediment depth. In this study, sediment cores were collected from both Daxingkai Lake and Xiaoxingkai Lake. By integrating metagenomic sequencing with P fractionation techniques, the research aims to (1) analyze vertical variation in bacterial and archaeal beta diversity along sediment depth and the differences between two lakes, (2) elucidate the vertical distribution characteristics of sedimentary P fractions and their inter-lake variations, and (3) determine the influence of P fraction dynamics on microbial beta diversity. The findings are expected to provide a scientific basis for eutrophication control and ecological restoration in Xingkai Lake and similar hydrologically connected lake-wetland systems.

## 2 Materials and methods

### 2.1 The study area

Xingkai Lake, located in southeastern of Heilongjiang province along the China-Russia border, consists of two parts, Daxingkai Lake and Xiaoxingkai Lake. The Xiaoxingkai Lake lies north of Daxingkai Lake and is connected to it via two floodgates. It covers an area of approximately 176 km^2^, with an average water depth of 2.5 m and a storage capacity of approximately 3.1 × 10^8^ m^3^ (Li et al., 2025). The Daxingkai Lake straddles the China-Russia border, with a total area of approximately 4,380 km^2^, of which the northern part (1,240 km^2^) belongs to China and the southern part (3,140 km^2^) belongs to Russia. Its average water depth is 6.6 m, and its storage capacity is about 153.3 × 10^8^ m^3^ (Li et al., 2025). The multi-year average air temperature in the Xingkai Lake region is 5.1 °C, and the mean annual precipitation is 734 mm, with summer (June to August) rainfall accounting for about 64% of the annual total. Prior to the 1850s, Xingkai Lake was minimally affected by human activity. In 1955, the Xingkai Lake State Farm was established, leading to the reclamation of large areas of land for agriculture. After 1980, adjustments in the industrial structure resulted in a significant expansion of rice cultivation. Since 2000, tourism in the Xingkai Lake area has developed rapidly.

In recent years, the spatial extent of algal blooms in Xingkai Lake has shown a continuous increase. Total phosphorus (TP) has been identified as the limiting factor for bloom outbreaks and the primary pollutant exceeding water quality standards in Xingkai Lake (Li et al., 2025). TP concentrations in the water of Daxingkai Lake and Xiaoxingkai Lake ranged from 0.020–0.250 mg/L and 0.020–0.240 mg/L, respectively, with mean concentrations of 0.080 mg/L and 0.071 mg/L. These levels represent a significant increase compared to the period 1993 ~ 1998, when mean TP concentrations were 0.036 mg/L and 0.040 mg/L, respectively (Li et al., 2025).

### 2.2 Sample collection and processing

**Figure 1:**
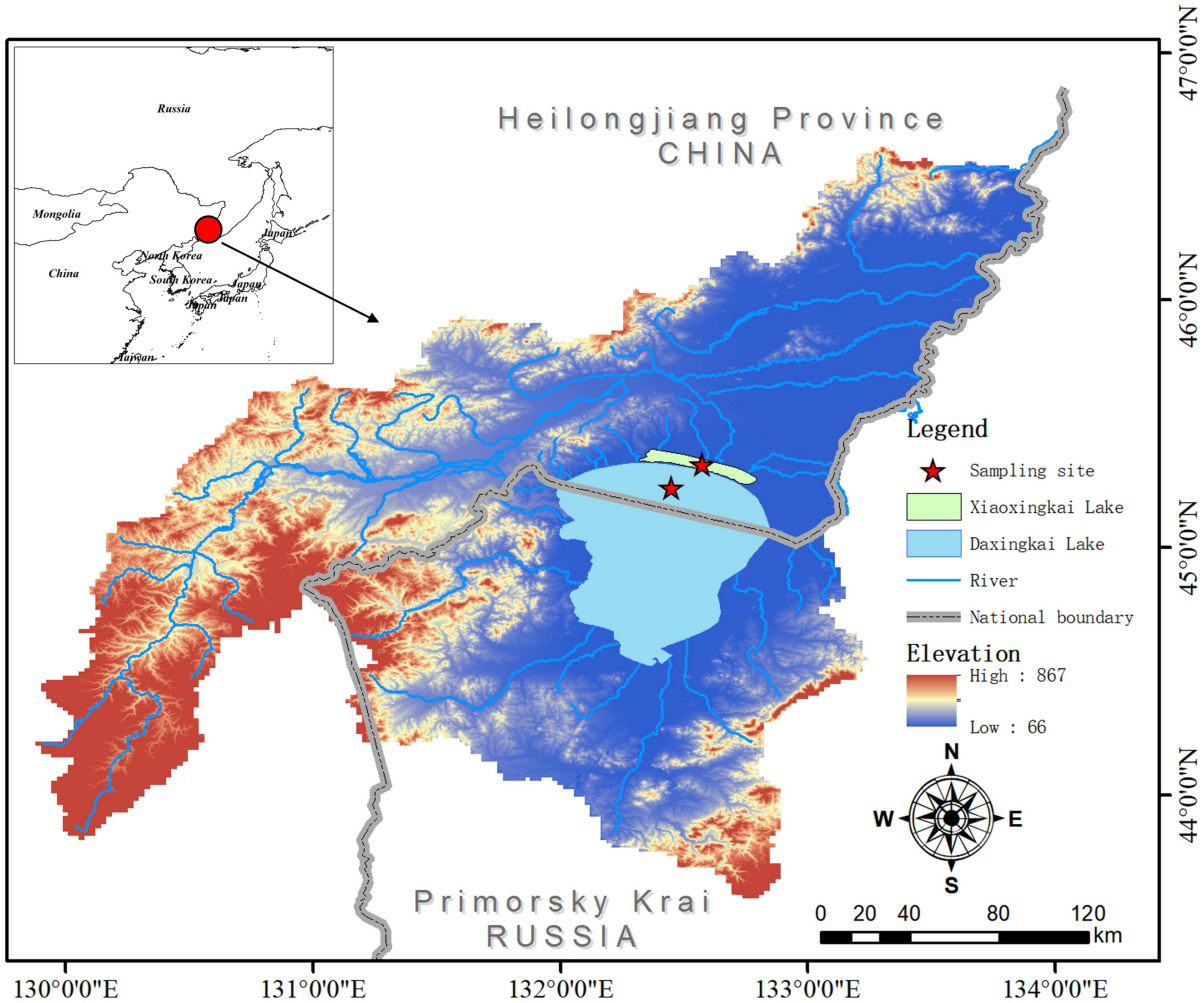
The distribution map of the study area and sampling point.

In order to investigate the sedimentary record of changes in nutrient loading and microbial communities, sediment cores were collected from both lake basins. In November 2024, a gravity corer fitted with a polyethylene column was used to retrieve six sediment cores from two representative sites in Daxingkai Lake (DXKL:132.450849 E, 45.244130 N) and Xiaoxingkai Lake (XXKL:132.576219 E, 45.338314 N) respectively (Figure 1). The depth of water at the sampling site was 7.1 m for DXKL and 2.3 m for XXKL, respectively. The cores were immediately segmented at 2-cm intervals. Each sediment segment was thoroughly homogenized using a sterile spatula and sub-sampled into two pre-labeled, sterile 50-mL centrifuge tubes. One set of samples was immediately placed on dry ice and subsequently stored at −80 °C for future molecular biological analysis. The other set was stored at −20 °C for subsequent physicochemical analysis, including determination of P fractions.

### 2.3 Analysis of sediment phosphorus fractions

Sediment P fractionation was performed using a sequential extraction procedure based on the widely applied Psenner fractionation scheme (Yuan et al., 2023). This method effectively separates sediment P into five operationally defined fractions reflecting different bioavailability and binding mechanisms: loosely adsorbed P (NH_4_Cl-P), reductant-soluble P (BD-P, mainly Fe-bound P), metal oxide-bound P (NaOH-P, mainly Al-bound P), calcium-bound P (HCl-P), and residual P (Res-P).

For each sediment sample, 0.20 g of freeze-dried material was sequentially extracted, and the detailed procedure was as follows: (1) NH_4_Cl-P: the sediment was shaken with 25 mL of 1 M NH_4_Cl for 0.5 h; (2) BD-P: the residue from step (1) was extracted with 25 mL of 0.11 M NaHCO_3_/Na_2_S_2_O_4_ solution for 1 h; (3) NaOH-P: the subsequent residue was shaken with 25 mL of 1 M NaOH for 16 h; (4) HCl-P: the remaining solid was then extracted with 25 mL of 0.5 M HCl for 16 h. All extractions were performed on a thermostatic shaker at 220 rpm. Following each extraction step, the suspension was centrifuged at 4000 rpm for 15 min. (5) Res-P: the final residue was combusted at 500 °C for 2 h, followed by shaking with 25 mL of 1 M HCl for 16 h. The resulting supernatant was collected after centrifugation and filtration. The supernatant was filtered through a 0.45 μm polytchersulfone membrane.

The PO_4_^3−^ concentration in all extracts, representing the inorganic P (Pi) of each fraction (i.e., NH_4_Cl-Pi, BD-Pi, NaOH-Pi and HCl-Pi), was quantified using the molybdate blue method (Murphy and Riley, 1962; Yuan et al., 2023; Xie et al., 2025). To determine the TP content of each fraction (i.e., NH_4_Cl-TP, BD-TP, NaOH-TP, HCl-TP), separate aliquots of the NH_4_Cl, BD, NaOH, and HCl extracts were digested with potassium persulfate at 120 °C for 0.5 h prior to PO_4_^3−^ measurement. The organic P (Po) content for each fraction (i.e., NH_4_Cl-Po, BD-Po, NaOH-Po, HCl-Po) was calculated as the difference between its TP and Pi (Po = TP - Pi). Sediment TP was calculated as the sum of NH_4_Cl-P, BD-P, NaOH-P, HCl-P, and Res-P.

### 2.4 High-throughput sequencing and bioinformatic analysis of sediment microorganisms

Metagenomic sequencing and subsequent analyses were conducted by Wekemo Tech Group Co., Ltd. (Shenzhen, China). Genomic DNA was extracted from 0.25 g of sediment using the cetyltrimethylammonium bromide method (Wang et al., 2021; Xie et al., 2025). DNA purity was assessed with a NanoDrop 2000 spectrophotometer (Thermo Scientific, Wilmington, DE, USA) by measuring the absorbance ratios at 260 nm and 280 nm. DNA concentration was quantified using a Qubit® 2.0 Fluorometer (Thermo Scientific, Wilmington, DE, USA). Sequencing libraries were prepared with the NEBNext Ultra II DNA Library Prep Kit (Illumina, USA) and sequenced on an Illumina NovaSeq platform (PE150 mode, Illumina, USA). Raw reads were Quality-controlled with FastQC software (Wang et al., 2021; Xie et al., 2023). Taxonomic profiling was performed using Kraken 2 with a customized reference database, and Bracken was used to estimate species abundance in each sample (Wang et al., 2021; Xie et al., 2023).

### 2.5 Data analysis

First, microbial abundances and functional genes were extracted and analyzed using the Wekemo Bioincloud Platform, with clustering heatmaps generated based on one-way ANOVA. Second, the “betapart” package in R(V4.5.1) was used to calculate total beta diversity and decompose it into species turnover and nestedness. Specifically, total beta diversity was quantified as the Sørensen dissimilarity index. Following the framework of Baselga, turnover was assessed by the Simpson coefficient, nestedness was calculated by the Nested coefficient. The relationship between sediment depth and microbial beta diversity in both lakes was then examined, and significance was assessed using the Mantel test. Third, variation partitioning analysis (VPA) was performed using the “vegan” package to determine the relative explanatory power of P fraction to explaining total beta diversity. Finally, bar charts illustrating the content and proportion of P fractions were created using Origin (V2024).

## 3 Results and discussion

### 3.1 Vertical characteristics of bacterial or archaeal beta diversity

**Figure 2:**
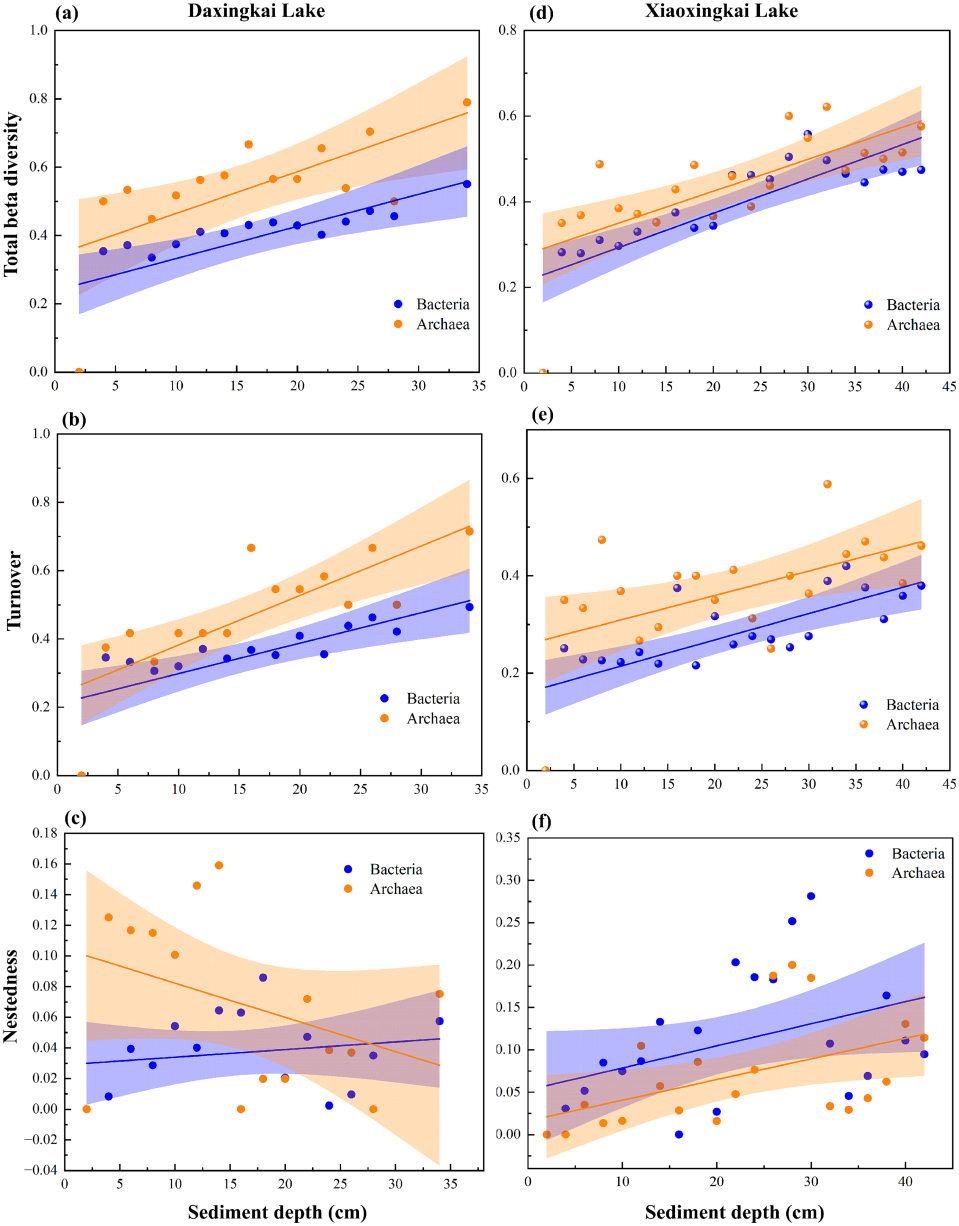
Vertical variation of bacteria and archaea beta diversity in Daxingkai Lake **(a–c)** and Xiaoxingkai Lake **(d–f)** with sediment depth.

Significant positive relationships were observed between sediment depth and multiple beta-diversity metrics for both bacterial and archaeal communities in the two lakes (Figure 2; Table 1). In Daxingkai Lake, bacterial total beta diversity and turnover increased significantly with sediment depth (total beta diversity: R^2^ = 0.544, *p* = 0.0017; turnover: R^2^ = 0.563, *p* = 0.0013). Archaea exhibited a stronger increase in species turnover along the depth gradient (R^2^ = 0.619, *p* = 0.0005). In contrast, the nestedness showed no significant relationship with sediment depth for either bacteria or archaea in this lake (R^2^ = 0.034, *p* = 0.5127), indicating that sediment depth explains only a small fraction of nestedness variation. Similarly, total beta diversity and species turnover of bacteria increased significantly with sediment depth in Xiaoxingkai Lake (total beta diversity: R^2^ = 0.665, *p* < 0.0001; turnover: R^2^ = 0.540, *p* = 0.0001). Archaeal total beta diversity also increased significantly with depth (R^2^ = 0.505, *p* = 0.0003), while archaeal turnover displayed a weaker but still significant relationship (R^2^ = 0.294, *p* = 0.0111). In contrast to Daxingkai Lake (R^2^ = 0.142, *p* = 0.1657), archaeal nestedness in Xiaoxingkai Lake demonstrated a significant positive correlation with sediment (R^2^ = 0.237, *p* = 0.0253). The regression slope reflects the rate of change in beta diversity per centimeter of sediment depth. Compared to Xiaoxingkai Lake (bacteria: slope = 0.0080; archaea: slope = 0.0075; Figure 2a), the total beta diversity of both bacterial and archaeal communities increased more rapidly with sediment depth in the Daxingkai Lake (bacteria: slope = 0.0094; archaea: slope = 0.0123; Figure 2d). A similar pattern was observed for turnover, with lower slopes in the Xiaoxingkai Lake (bacteria: slope = 0.0054; archaea: slope = 0.0050; Figure 2e) than in the Daxingkai Lake (bacteria: slope = 0.0089; archaea:slope = 0.0145; Figure 2b).

**Table 1:** Statistical analysis of bacteria and archaea correlation parameters in Daxingkai Lake and Xiaoxingkai Lake.

Lake	Beta diversity	Bacteria			Archaea		
		R^2^	P	Slope	R^2^	P	Slope
Daxingkai Lake	Total beta diversity	0.544	0.0017	0.0094	0.441	0.0070	0.0123
	Turnover	0.563	0.0013	0.0089	0.619	0.0005	0.0145
	Nestedness	0.034	0.5127	0.0005	0.142	0.1657	−0.0022
Xiaoxingkai Lake	Total beta diversity	0.665	<0.0001	0.0080	0.505	0.0003	0.0075
	Turnover	0.540	0.0001	0.0054	0.294	0.0111	0.0050
	Nestedness	0.171	0.0620	0.0026	0.237	0.0253	0.0024

**Figure 3:**
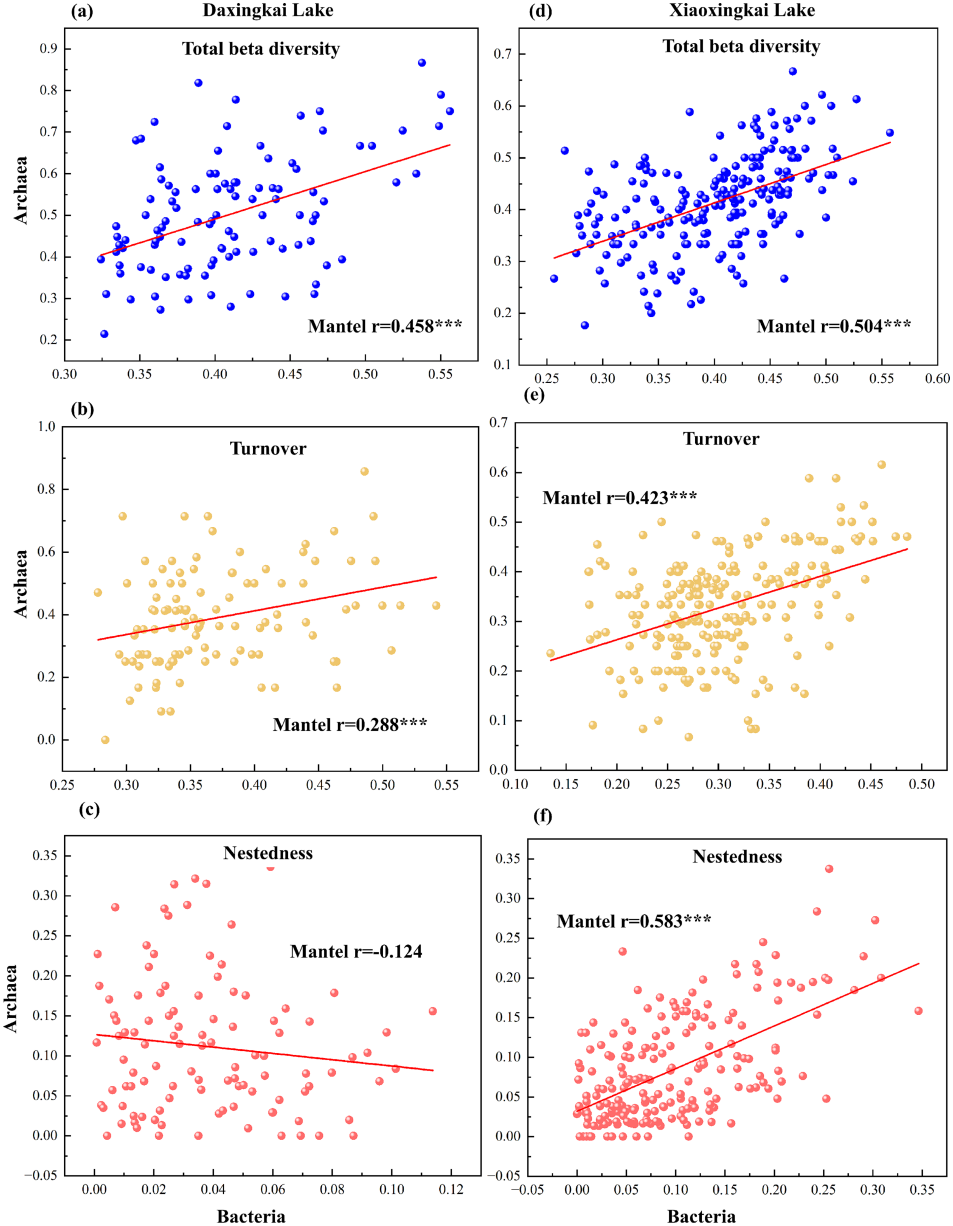
The correlation between bacteria and archaea beta diversity in Daxingkai Lake **(a–c)** and Xiaoxingkai Lake **(d–f)** with sediment depth. *p ≤ 0.05; **p < 0.01; *** *p* < 0.001.

Mantel tests revealed significant positive correlations between bacterial and archaeal communities in total beta diversity in both lakes (DXKL: Mantel *r* = 0.458, *p* < 0.001; XXKL: Mantel *r* = 0.504, *p* < 0.001; Figures 3a,d). A similar positive relationship was observed for the species turnover component (DXKL: Mantel *r* = 0.423, *p* < 0.001; XXKL: Mantel *r* = 0.288, *p* < 0.01; Figures 3b,e). Notably, the nestedness component of bacterial and archaeal communities showed a strongly significant positive correlation in Xiaoxingkai Lake (Mantel *r* = 0.583, *p* < 0.001; Figure 3f), whereas only a weak and non-significant negative correlation was detected in Daxingkai Lake (Mantel *r* = −0.124, *p* > 0.05; Figure 3c). The significant positive correlation between bacteria and archaea in total beta diversity and species turnover across the two lakes suggests a parallel pattern of community variability along the sediment depth gradient.

The consistent increase in total beta diversity and species turnover with sediment depth across both lakes supports the prevailing view that species replacement dominates microbial community variation along vertical environmental gradients in freshwater sediments (Baselga, 2010; Yuan et al., 2023). Such depth-related patterns are widely attributed to intensified environmental filtering and niche differentiation under increasingly heterogeneous sedimentary conditions (Martiny et al., 2011; Yuan et al., 2024). Notably, the faster increase in archaeal turnover rates in Daxingkai Lake compared to Xiaoxingkai Lake indicates greater sensitive of archaeal communities to depth-driven environmental shifts (Yuan et al., 2023; Yu et al., 2023). The contrasting patterns of nestedness between the two lakes highlight that while species turnover is the dominant component overall, nestedness may become significant under specific environmental contexts (Legendre, 2014; Yuan et al., 2024). This difference likely results from stronger environmental filtering and selective species loss in the shallower, more disturbed Xiaoxingkai Lake (2.5 m), whereas the deeper Daxingkai Lake (6.6 m) exhibits a more continuous vertical gradient where species turnover prevails.

### 3.2 Vertical characteristics of phosphorus fraction

**Figure 4:**
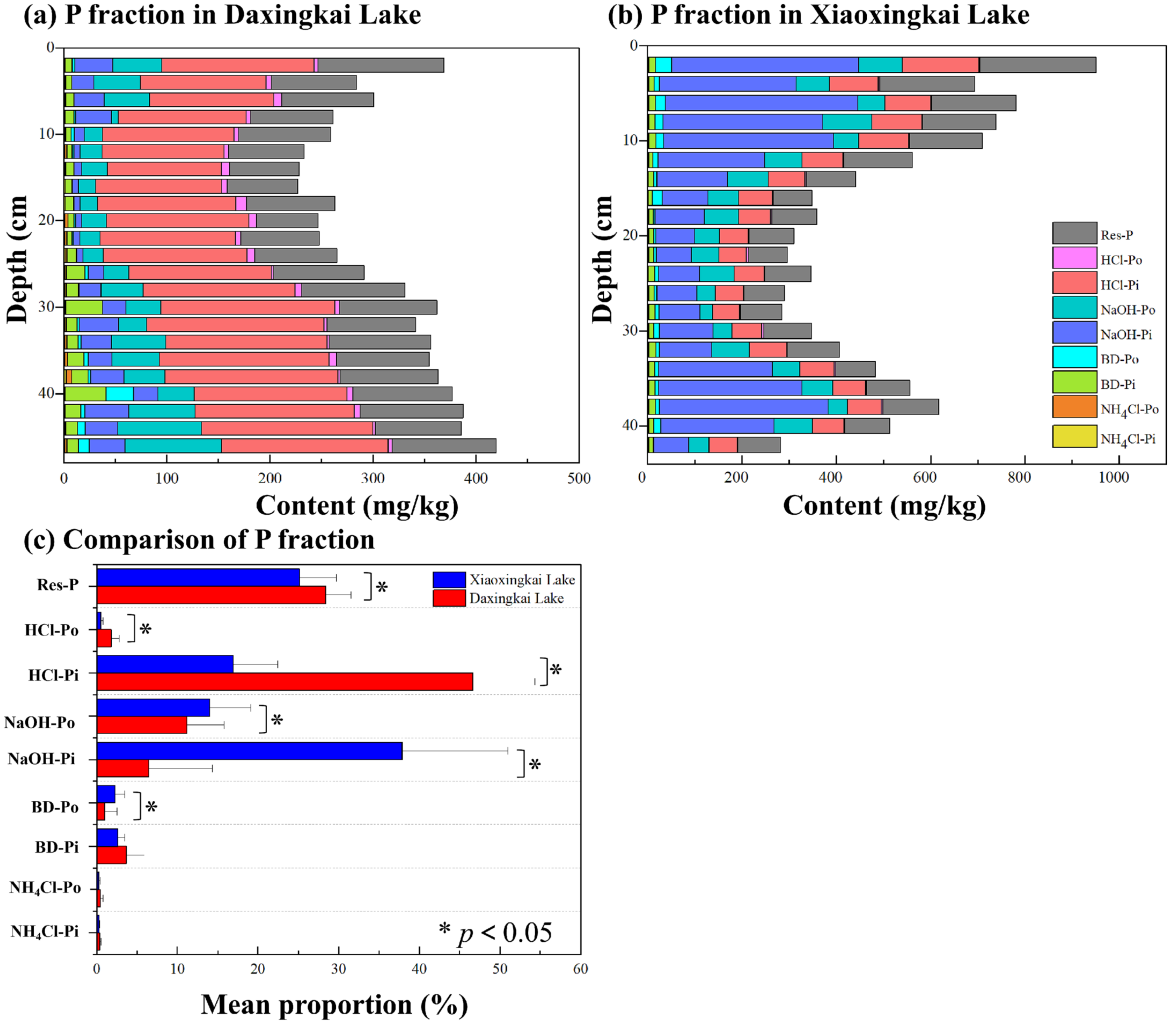
Vertical distribution of P fractions **(a,b)** and comparison of their mean proportion **(c)** in Daxingkai Lake and Xiaoxingkai Lake.

The vertical profiles of P fractions exhibited distinct patterns between the two lakes (Figure 4). In Daxingkai Lake, TP displayed an initial decrease followed by an increase with depth, ranging from 227.0 to 419.5 mg kg^−1^, with a mean value of 311.0 mg kg^−1^ (Figure 4a). The minimum occurred at a depth of 15 ~ 16 cm. In Xiaoxingkai Lake, TP showed a more complex trend, decreasing initially, then increasing and decreasing again with sediment depth (Figure 4b). Its concentration varied from 281.8 to 950.5 mg kg^−1^, averaging 491.3 mg kg^−1^, with the lowest value observed at 41 ~ 42 cm (Figure 4b). The composition of P fractions also differed notably between the lakes (Figure 4c). In Daxingkai Lake, the dominant P forms were HCl-Pi (46.63%), Res-P (28.41%) and NaOH-Po (11.18%), while in Xiaoxingkai Lake, the dominant P fractions were NaOH-Pi (37.89%), Res-P(25.14%) and HCl-Pi(16.95%). HCl-Pi and Res-P showed an increasing trend with sediment depth in Daxingkai Lake, while in Xiaoxingkai Lake, NaOH-Pi decreased significantly below 20 cm, and then increased. Compared to Xiaoxingkai Lake, Daxingkai Lake contained a higher proportion of HCl-Pi, HCl-Po and Res-P, but a lower proportion of NaOH-Pi, BD-Po and NaOH-Po.

The distinct vertical profiles of P fractions observed between Daxingkai and Xiaoxingkai Lakes may reflect differences in historical sedimentation characteristics, redox conditions, and external P loading. The dominance of HCl-Pi and Res-P in Daxingkai Lake suggests a greater proportion of stable, calcium-bound and refractory P, where calcium ions facilitate the precipitation of inert P forms (Yuan et al., 2024). In contrast, the higher proportion of NaOH-Pi in Xiaoxingkai Lake indicates a greater abundance of metal oxide-bound P, which is more susceptible to release under anoxic conditions and may contribute to a higher potential for internal P loading (Xie et al., 2023; Yuan et al., 2023). These compositional differences in P fractions are critical for understanding the bioavailability of sediment P and its regulatory role in shaping microbial community assembly and succession (Yuan et al., 2023).

Based on ^210^Pb and ^137^Cs dating of sediment cores from Xingkai Lake (Li et al., 2025), the sedimentary record was divided into three stages: (1) natural background stage (>30 cm, pre-1955), with minimal human impact and oligotrophic-mesotrophic conditions; (2) development stage (15–30 cm, 1955–2000), when agricultural reclamation increased external P input, and the lake began transitioning to eutrophic status; and (3) eutrophic stage (<15 cm, 2000-present), characterized by intensified eutrophication, frequent algal blooms, and increased agricultural and tourism inputs. In XXKL, the eutrophic stage showed a substantial increase in NaOH-Pi content, bacterial total beta diversity, and archaeal nestedness compared to the natural background stage (Figure 2; Figure 4). In contrast, DXKL exhibited only a modest increase in HCl-Pi content and bacterial total beta diversity, with no significant change in archaeal nestedness (Figure 2; Figure 4). These results indicate that P transformation characteristics differ among eutrophication stages, and that microbial beta diversity and its components can sensitively reflect the progression of lake eutrophication. In XXKL, NaOH-Pi accounts for 37.89% of sedimentary P (Figure 4). This fraction, primarily bound to Fe/Mn oxides, is highly susceptible to anoxic release and serves as a key P source fueling algal blooms (Yuan et al., 2023; Woolway et al., 2024). Additionally, XXKL sediments are enriched in phosphate-solubilizing bacteria and methanogenic archaea, whose metabolic activities further promote P release, resulting in a high risk of internal P loading. Conversely, DXKL sediments are dominated by HCl-Pi (46.63%) and Res-P (28.41%), which are resistant to release under normal environmental conditions, indicating a low risk of internal P loading.

### 3.3 Relationship between microbial community structure and phosphorus fraction

The top 20 bacterial and top 5 archaeal genera ranked by relative abundance were selected for analysis (Supplementary Figures S1, S2). Compared with Daxingkai Lake, bacterial genera including *Lacipirellula*, *Mycobacterium*, *Desulfobacterium* and *Bacillus* were significantly enriched in Xiaoxingkai Lake, while *Acidobacterium*, *Nitrospira* and *Mycolicibacter* were significantly depleted (Supplementary Figure S3). For archaea, *Methanobacterium*, *Methanothrix*, *Methanosarcina*, *Haloferax* and *Halorubrum* showed significantly higher abundance in Xiaoxingkai Lake, whereas *Nitrososphaera*, *Candidatus_Nitrosotalea*, *Methanobrevibacter* and *Halobiforma* were significantly more abundant in Daxingkai Lake (Supplementary Figure S3).

**Figure 5:**
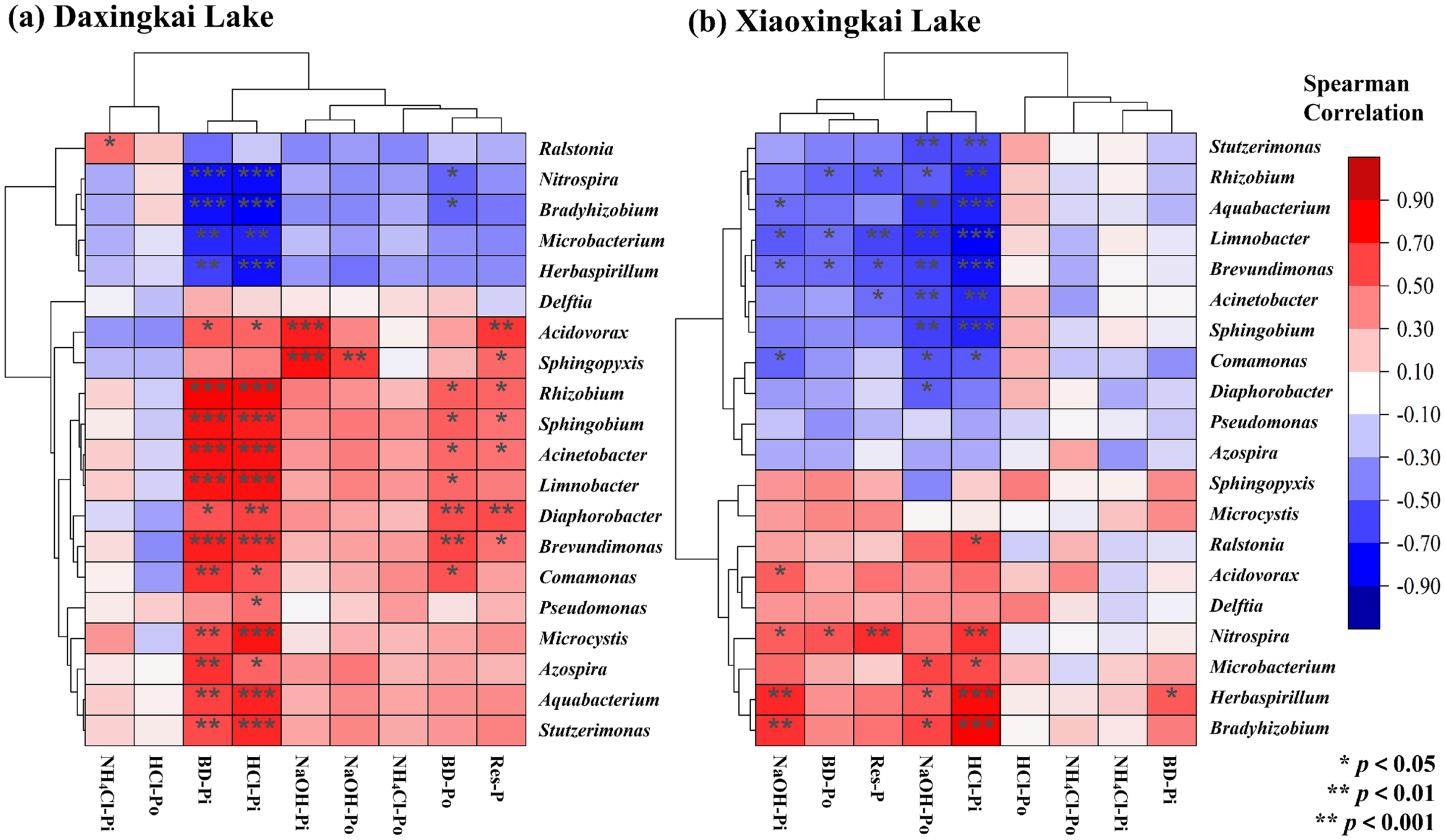
The correlation analysis of bacterial communities and P fractions at the genus level in Daxingkai Lake **(a)** and Xiaoxingkai Lake **(b)**.

Correlation analysis revealed a strong and taxon-specific association between sediment P fractions and bacterial community composition in both lakes (Figure 5). With regard to the Daxingkai Lake, several P fractions, including BD-Pi, HCl-Pi, BD-Po and Res-P showed significant correlations with multiple dominant bacterial genera (*p* < 0.05; Figure 5a). These fractions were negatively correlated with genera such as *Nitrospira* and *Bradyrhizobium*, but positively correlated with *Acidovorax*, *Rhizobium* and *Sphingobium* (*p* < 0.05; Figure 5a). NH_4_Cl-P was positively correlated only with *Ralstonia*, while NaOH-Pi and NaOH-Po showed a significant positive correlation with *Sphingopyxis* (*p* < 0.05; Figure 5a). As for the Xiaoxingkai Lake, NaOH-Pi, BD-Po, Res-P, NaOH-Po and HCl-Po were significantly correlated with various dominant genera (*p* < 0.05; Figure 5b). These fractions correlated negatively with *Limnobacter* and *Brevundimonas*, but positively correlated with *Nitrospira* and *Herbaspirillum* (*p* < 0.05; Figure 5b). Notably, BD-Pi was positively correlated with *Herbaspirillum* (*p* < 0.05; Figure 5b). Several bacterial genera, including *Microbacterium*, *Herbaspirillum*, *Azospir*a and *Stutzerimonas*, exhibited opposite correlation trends with the same P fractions between the two lakes, indicating divergent ecological responses to P speciation in different sedimentary environments.

**Figure 6:**
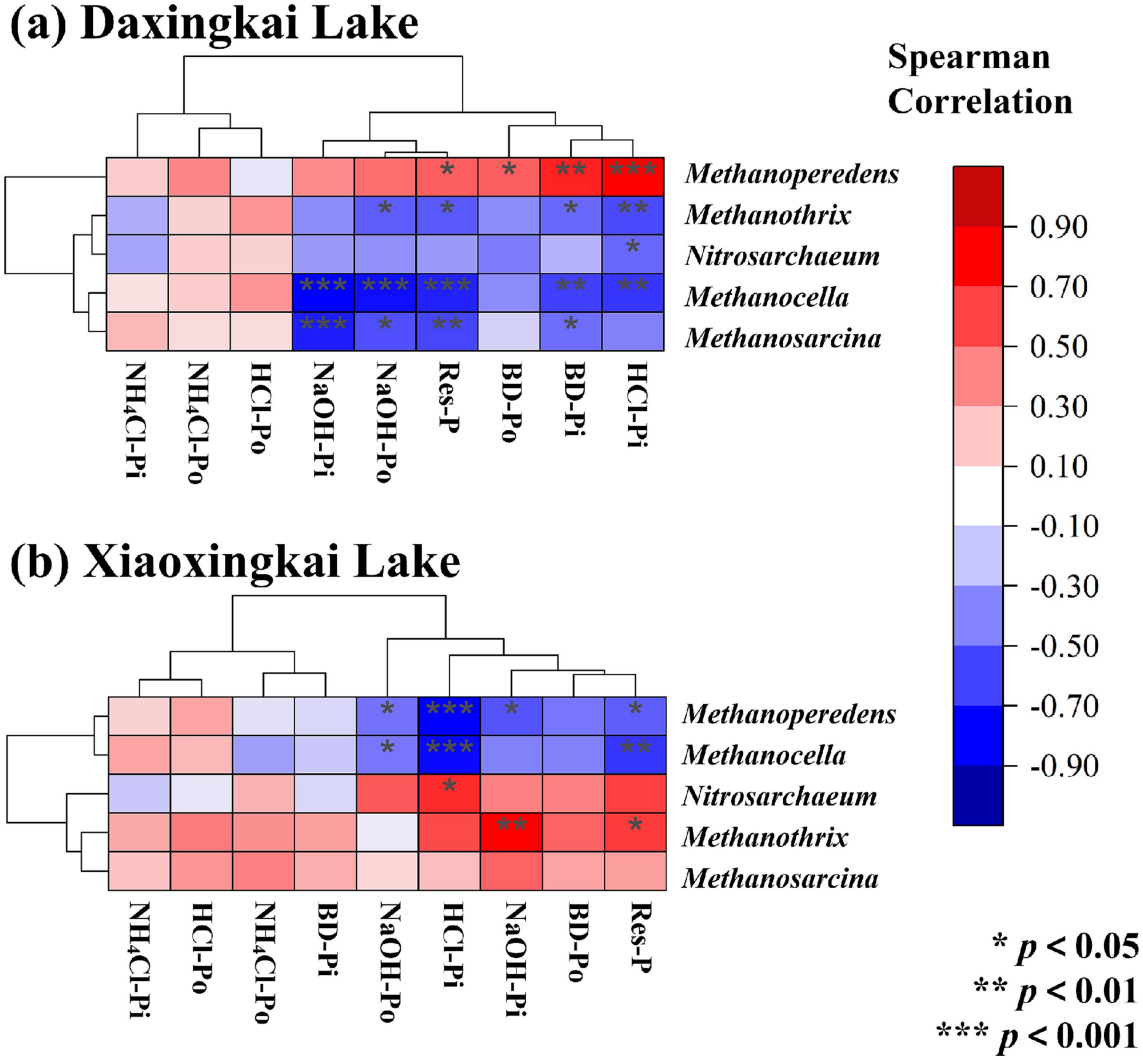
The correlation analysis of archaeal communities and P fractions at the genus level in Daxingkai Lake **(a)** and Xiaoxingkai Lake **(b)**.

Similarly, significant correlations were observed between sediment P fractions and archaeal community composition in the two lakes (*p* < 0.05; Figure 6). In Daxingkai Lake, several P fractions, including NaOH-Pi, NaOH-Po, Res-P, BD-Pi and HCl-Pi, showed significant positive correlations with *Methanoperedens* (*p* < 0.05; Figure 6a). Moreover, these fractions were significantly negatively correlated with typical methanogenic genera, such as *Methanothrix*, *Methanocella*, and *Methanosarcina* (*p* < 0.05; Figure 6a). In contrast, in Xiaoxingkai Lake, NaOH-Po, HCl-Pi, NaOH-Pi and Res-P exhibited significant negative correlations with both *Methanoperedens* and *Methanocella* (*p* < 0.05; Figure 6b). Furthermore, HCl-Pi was positively correlated with *Nitrosarchaeum*, and NaOH-Pi and Res-P were negatively correlated with *Methanothrix* (*p* < 0.05; Figure 6b).

The correlations between P fractions and microbial communities observed in Daxingkai and Xiaoxingkai Lakes highlight the ecological importance of P resource partitioning in shaping bacterial and archaeal assemblages in lacustrine sediments. The differential utilization of P compounds by microbial taxa suggests that such partitioning may alleviate competitive pressure and promote niche differentiation among co-occurring microorganisms (Ceulemans et al., 2017; Yuan et al., 2024). The stronger associations with inorganic P fractions (e.g., BD-Pi, HCl-Pi, NaOH-Pi) observed in both lakes align with previous findings that inorganic P fractions exert greater selective pressure on microbial community composition than organic P fractions, particularly in eutrophic lake systems (Yuan et al., 2023). The opposite correlation trends exhibited by several bacterial genera (e.g., *Herbaspirillum*, *Microbacterium*) toward the same P fractions between the two lakes indicate that the ecological effects of P speciation are highly context-dependent, modulated by lake-specific environmental factors such as sediment redox status, metal ion availability (Fe, Mn, Ca), and historical pollution legacy (Yuan et al., 2024). Furthermore, the significant correlations between archaeal genera (e.g., *Methanopereden*s, *Methanothrix*) and both Pi and Po fractions suggest that archaeal communities may play an active role in organic P mineralization (Yuan et al., 2023; Baker et al., 2020).

*Nitrospira* requires substantial ATP for ammonia and nitrite oxidation, making it sensitive to Pi availability (Song et al., 2024; Woolway et al., 2024). Anoxic conditions inhibit its nitrification activity and reduce energy production efficiency, resulting in a negative correlation with BD-Pi. *Methanoperedens* is a key archaeal lineage mediating nitrate-dependent anaerobic methane oxidation (n-DAMO) (R). In DXKL, *Methanoperedens* shows significant positive correlations with NaOH-Pi and HCl-Pi. because n-DAMO promotes dissolution of calcium-bound P and the archaea accumulate Pi during growth. In contrast, *Methanoperedens* exhibits significant negative correlations with Pi in XXKL. This discrepancy is likely due to higher external P inputs in XXKL, which stimulate methanogens growth and increase methane concentrations. Consequently, *Methanoperedens* abundance is primarily limited by methane availability rather than P concentration, and high Pi levels inhibit key n-DAMO enzyme activities. Furthermore, *Herbaspirillum* correlates negatively with BD-Pi in DXKL but positively with BD-Pi in XXKL. *Herbaspirillum* is a bacterial genus capable of both nitrogen fixation and P solubilization, exhibiting highly flexible metabolic strategies (Song et al., 2024; Woolway et al., 2024). In DXKL, it primarily acquires P through solubilization, thus correlating positively with insoluble P fractions. In XXKL, where Pi is abundant, its P-solubilizing genes are downregulated, and it instead utilizes readily available environmental P, resulting in a positive correlation with the easily released BD-Pi. This explains why the same microbial genus can show opposing P response patterns in different environments.

### 3.4 Relationship between P fractions and the beta diversity of bacterial and archaeal communities

**Figure 7:**
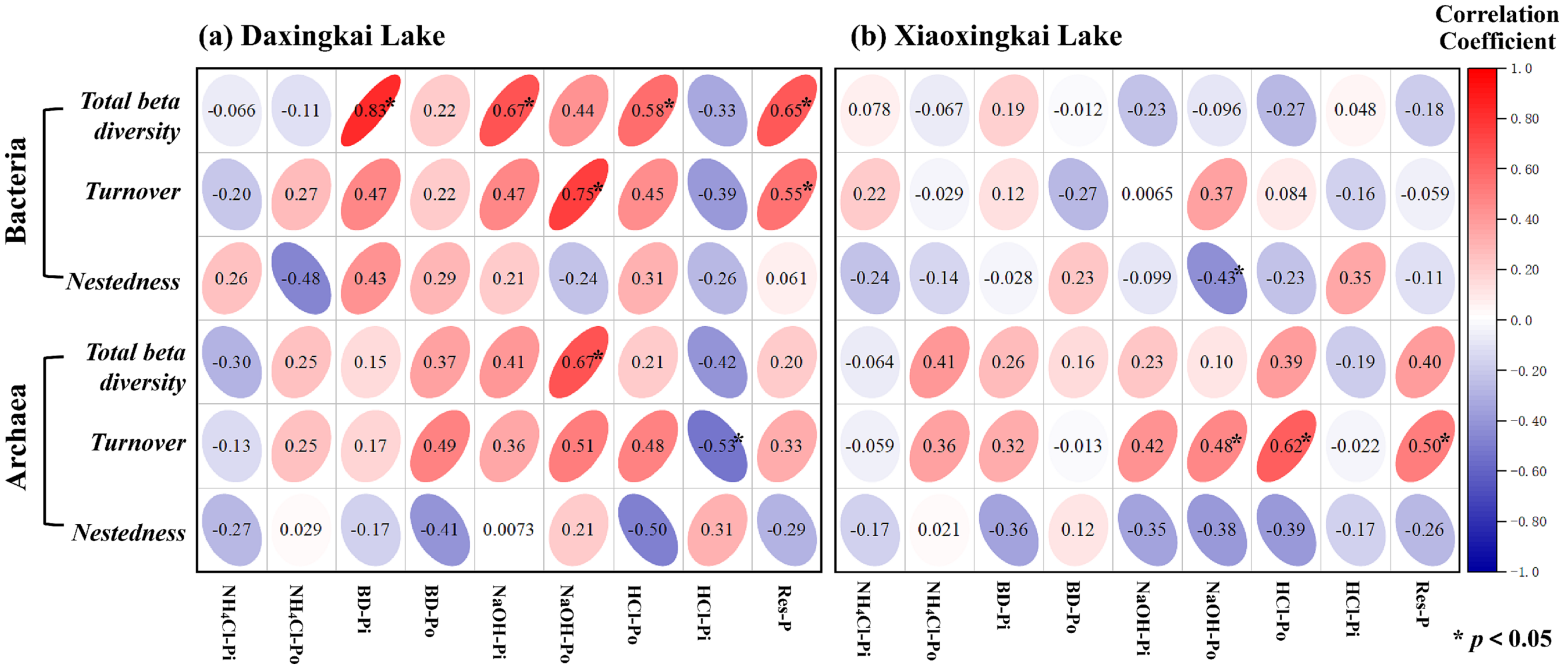
The correlation between P fractions and bacteria or archaea total beta diversity and their components in Daxingkai Lake **(a)** and Xiaoxingkai Lake **(b)**.

The relative importance of P fractions in explaining beta diversity varied between microbial domains and lakes (Figure 7). In Daxingkai Lake, bacterial beta diversity was strongly associated with BD-Pi, NaOH-Pi, HCl-Po and Res-P, which showed relatively high standardized regression coefficients in explaining total beta diversity (R = 0.83, 0.67, 0.58 and 0.65, respectively; *p* < 0.05). NaOH-Po and Res-P also showed high coefficients in relation to species turnover (R = 0.75, 0.55; *p <* 0.05). In contrast, NH_4_Cl-Po and BD-Pi exhibited higher coefficients for nestedness (R = −0.48 and 0.43, respectively), although these were not statistically significant (*p* > 0.05). As for the Xiaoxingkai Lake, bacterial beta diversity showed numerically higher associations with NH_4_Cl-Pi, whereas other fractions such as BD-Pi, NaOH-Pi, and Res-P exhibited weak and non-significant associations (Figure 7b). Only nestedness demonstrated a significant negative correlation with NaOH-Po (R = −0.43; *p* < 0.05) (Figure 7b). For archaea, NaOH-Po and HCl-Pi displayed high coefficients in explaining total beta diversity and turnover in Daxingkai Lake, whereas HCl-Po and Res-P were more strongly associated with these diversity components in Xiaoxingkai Lake (Figure 7).

**Figure 8:**
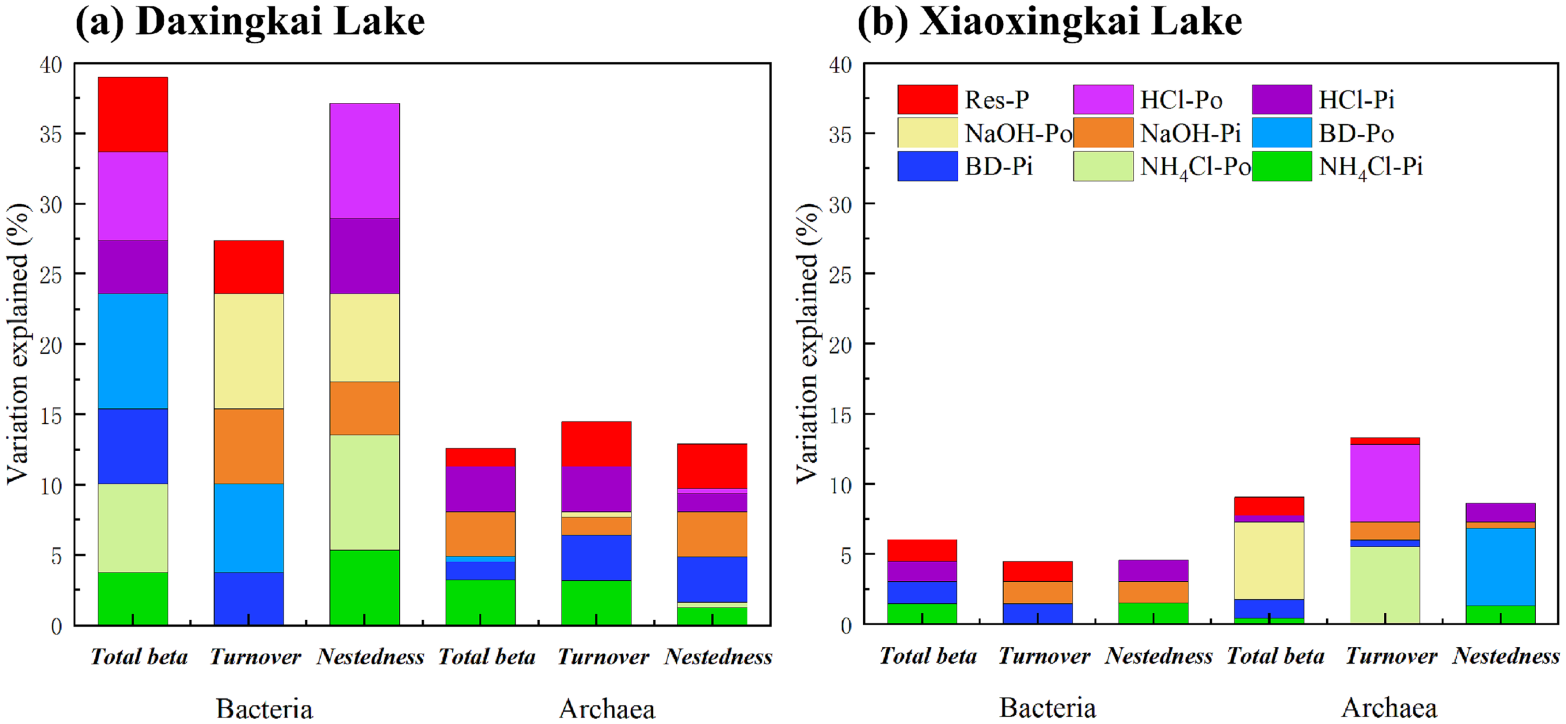
Relative importance of phosphorus fractions in explaining variation of bacterial and archaeal beta diversity in Daxingkai Lake **(a)** and Xiaoxingkai Lake **(b)**.

The independent contributions of specific P fractions to microbial beta diversity, derived from variation partitioning analysis, revealed contrasting patterns between the two lakes (Figure 8). With regard to the Daxingkai Lake, organic P fractions explained the largest proportion of variation in bacterial communities, accounting for 20.8, 14.5 and 22.7% of the variation in total beta diversity, species turnover and nestedness, respectively (Figure 8a). Specifically, BD-Po showed the highest contribution to total beta diversity, NaOH-Po was the primary driver of species turnover, while HCl-Po and NH_4_Cl-Po exerted the greatest influence on the nestedness component (Figure 8a). Conversely, for archaea in Daxingkai Lake, inorganic P fractions explained a greater proportion of variation, accounting for 10.9, 10.9 and 9.0% in total beta diversity, turnover and nestedness, with NH_4_Cl-Pi, NaOH-Pi, and BD-Pi showing relatively similar levels of contribution (Figure 8a).

In Xiaoxingkai Lake, the pattern differed from that observed in Daxingkai Lake. For bacteria, inorganic P fractions showed the highest pure explanatory power, accounting for 4.5, 3.0 and 4.6% of the variation in total beta diversity, turnover and nestedness (Figure 8b), with NH_4_Cl-Pi, NaOH-Pi, and BD-Pi being major contributors. For archaea in this lake, organic P fractions played a dominant role, explaining 5.6, 11.1, and 5.6% of the variation in total beta diversity, turnover, and nestedness, respectively (Figure 8b). Among them, NaOH-Po contributed most to total beta diversity, HCl-Po and NH_4_Cl-Po were key for species turnover, and BD-Po was the primary factor for nestedness (Figure 8b).

The divergent contributions of organic and inorganic P fractions to bacterial and archaeal beta diversity across Daxingkai and Xiaoxingkai Lakes highlight the context-dependent nature of P resource partitioning in driving microbial community assembly. These findings align with previous observations that species turnover, rather than nestedness, dominates beta diversity patterns in freshwater sediments, and that deterministic processes such as environmental selection by P fractions play a central role in shaping microbial community variation (Martiny et al., 2011; Yuan et al., 2023). The contrasting dominance of organic P fractions for bacterial beta diversity in Daxingkai Lake versus inorganic P fractions in Xiaoxingkai Lake underscores that the relative bioavailability and ecological impact of different P forms are modulated by lake-specific conditions (Yuan et al., 2024). The stronger explanatory power of inorganic P fractions for archaeal beta diversity in Daxingkai Lake, and organic P fractions in Xiaoxingkai Lake, further supports the notion that archaeal communities may exhibit taxon-specific dependencies on P forms, particularly in relation to anaerobic processes such as organic matter mineralization and methane cycling (Baker et al., 2020; Yuan et al., 2023). Collectively, these results emphasize that partitioning beta diversity into turnover and nestedness components, coupled with detailed characterization of P fractions, provides a robust framework for unraveling the mechanisms underlying microbial community succession in lacustrine ecosystems (Legendre, 2014; Yuan et al., 2024).

## 4 Conclusion

This study reveals that species turnover consistently dominates the vertical beta diversity patterns of both bacterial and archaeal communities in the sediments of two hydrologically connected twin lakes. However, nestedness emerges as a significant component under specific conditions, especially for archaea in the shallower and more anthropogenically influenced Xiaoxingkai Lake. Notably, organic and inorganic P fractions play divergent roles in shaping microbial beta diversity. Organic P fractions are more strongly associated with bacterial beta diversity in the Daxingkai Lake, whereas inorganic P fractions exert stronger influence on bacteria in the Xiaoxingkai Lake, with the opposite pattern observed for archaea. These contrasting findings suggest that P resource partitioning may act as an important factor structuring sediment microbial communities. This study provides a basis for developing microbial beta diversity indicators to support eutrophication assessment and sediment management in hydrologically connected lake systems. Future studies should incorporate multi-site sampling across each lake basin to better resolve the influence of whole-lake spatial heterogeneity and additional physicochemical factors on microbial beta diversity, and should also conduct functional gene analysis to identify the taxonomic groups that directly mediate P transformation processes.

## Data Availability

The metagenomic sequence data have been deposited in the NCBI SRA, under BioProject accession number PRJNA1469417 (https://www.ncbi.nlm.nih.gov/bioproject/1469417). Further inquiries can be directed to the corresponding author.
