# Supplementary material for: Effect of phosphorus fraction in shaping bacterial and archaeal community succession in the largest hydrologically connected lake of Northeast Asia
Source: Front Microbiol. 2026 Jul 28;17:1844785. doi: 10.3389/fmicb.2026.1844785 (PMC13475343; doi:10.3389/fmicb.2026.1844785)
Supplement: Supplementary file 1 [file Supplementary_file_1.DOCX]

Supplementary Information

for

**Effect of phosphorus fraction in shaping bacterial and archaeal community succession in the largest hydrologically connected lake of Northeast Asia**

Zijian Xie^a^, Xinghong Liu^a,b,1^, Bo Bo^a^, Weiwei Wei^a^, Chunhua Li^a,*^, Chun Ye^a,*^

^a^ Key Laboratory of Drinking Water Source Protection of the Ministry of Ecology and Environment, National Engineering Laboratory for Lake Pollution Control and Ecological Restoration, Chinese Research Academy of Environmental Sciences, Beijing, 100012, China

^b^ College of Environmental and Safety Engineering, Shenyang University of Chemical Technology, Shenyang,110142, China

Corresponding author:

Chun Ye (E-mail: yechun@craes.org.cn; 010-84915191)

Chunhua Li (E-mail: lich@craes.org.cn; 010-84915191)

Key Laboratory of Drinking Water Source Protection of the Ministry of Ecology and Environment, National Engineering Laboratory for Lake Pollution Control and Ecological Restoration, Chinese Research Academy of Environmental Sciences, Beijing 100012, China

**Figure S1.** The dominant bacterial phyla and genera in sediment cores of Daxingkai Lake and Xiaoxingkai Lake.

**Figure S2.** The dominant archaeal phyla and genera in sediment cores of Daxingkai Lake and Xiaoxingkai Lake.

**Figure S3.** Volcano plot of differential abundant bacterial and archaeal genera between Daxingkai Lake and Xiaoxingkai Lake sediments.

**Table S1.** The quality of metagenomic sequencing data output in Xingkai Lake.

| Sample ID | InsertSize  (bp) | SeqStrategy | RawReads  (#) | Raw Base  (GB) | %GC | Raw Q20  (%) | Raw Q30  (%) | Clean Reads  (#) | Cleaned  (%) | Clean Q20  (%) | Clean Q30  (%) |
| --- | --- | --- | --- | --- | --- | --- | --- | --- | --- | --- | --- |
| DXKL01 | 350 | (150:150) | 24532749 | 7.36 | 64 | 97.68 | 93.67 | 23373566 | 95.27 | 98.89 | 96.05 |
| DXKL02 | 350 | (150:150) | 29644064 | 8.89 | 64 | 97.62 | 93.17 | 28210754 | 95.16 | 98.72 | 95.43 |
| DXKL03 | 350 | (150:150) | 23642695 | 7.09 | 61 | 97.64 | 93.21 | 22280213 | 94.24 | 98.71 | 95.39 |
| DXKL04 | 350 | (150:150) | 26212940 | 7.86 | 62 | 97.65 | 93.39 | 24986843 | 95.32 | 98.78 | 95.62 |
| DXKL05 | 350 | (150:150) | 21841477 | 6.55 | 62 | 97.64 | 93.23 | 20808376 | 95.27 | 98.72 | 95.44 |
| DXKL06 | 350 | (150:150) | 30752714 | 9.23 | 61 | 97.98 | 94.21 | 29452735 | 95.77 | 98.96 | 96.20 |
| DXKL07 | 350 | (150:150) | 23022720 | 6.91 | 62 | 97.72 | 93.47 | 21915557 | 95.19 | 98.8 | 95.68 |
| DXKL08 | 350 | (150:150) | 26823031 | 8.05 | 62 | 97.79 | 93.77 | 25594583 | 95.42 | 98.85 | 95.90 |
| DXKL09 | 350 | (150:150) | 19823532 | 5.95 | 61 | 97.89 | 93.92 | 18924242 | 95.46 | 98.85 | 95.93 |
| DXKL10 | 350 | (150:150) | 26891968 | 8.07 | 56 | 98.96 | 96.86 | 26005724 | 96.70 | 99.54 | 98.14 |
| DXKL11 | 350 | (150:150) | 26991012 | 8.10 | 60 | 98.19 | 94.82 | 25879920 | 95.88 | 99.09 | 96.64 |
| DXKL12 | 350 | (150:150) | 25362552 | 7.61 | 55 | 98.89 | 96.70 | 24421425 | 96.29 | 99.55 | 98.16 |
| DXKL13 | 350 | (150:150) | 29852821 | 8.96 | 59 | 95.11 | 88.21 | 20879101 | 69.94 | 99.00 | 96.42 |
| DXKL14 | 350 | (150:150) | 26265215 | 7.88 | 52 | 97.84 | 94.33 | 23182919 | 88.26 | 99.59 | 98.32 |
| DXKL17 | 350 | (150:150) | 22931680 | 6.88 | 50 | 98.80 | 96.56 | 21111750 | 92.06 | 99.61 | 98.39 |
| XXKL01 | 350 | (150:150) | 21449938 | 6.43 | 62 | 97.71 | 93.50 | 17182252 | 80.10 | 98.88 | 95.99 |
| XXKL02 | 350 | (150:150) | 29070967 | 8.72 | 61 | 98.07 | 94.35 | 27875557 | 95.89 | 98.96 | 96.19 |
| XXKL03 | 350 | (150:150) | 20132673 | 6.04 | 62 | 97.96 | 94.17 | 19168853 | 95.21 | 98.95 | 96.17 |
| XXKL04 | 350 | (150:150) | 26395908 | 7.92 | 61 | 97.91 | 94.15 | 25289327 | 95.81 | 98.94 | 96.23 |
| XXKL05 | 350 | (150:150) | 27389080 | 8.22 | 62 | 97.62 | 93.30 | 26059800 | 95.15 | 98.76 | 95.57 |
| XXKL06 | 350 | (150:150) | 31588292 | 9.48 | 63 | 97.84 | 93.93 | 30116171 | 95.34 | 98.92 | 96.08 |
| XXKL07 | 350 | (150:150) | 31830786 | 9.55 | 62 | 97.88 | 93.94 | 30389759 | 95.47 | 98.88 | 95.98 |
| XXKL08 | 350 | (150:150) | 28700914 | 8.61 | 62 | 97.76 | 93.60 | 27359096 | 95.32 | 98.83 | 95.80 |
| XXKL09 | 350 | (150:150) | 26976718 | 8.09 | 59 | 98.12 | 94.45 | 25922998 | 96.09 | 98.96 | 96.21 |
| XXKL10 | 350 | (150:150) | 27973026 | 8.39 | 59 | 98.27 | 94.64 | 26513742 | 94.78 | 99.03 | 96.33 |
| XXKL11 | 350 | (150:150) | 26806872 | 8.04 | 55 | 98.52 | 95.63 | 25865402 | 96.49 | 99.24 | 97.12 |
| XXKL12 | 350 | (150:150) | 24406265 | 7.32 | 52 | 98.57 | 95.67 | 23600319 | 96.70 | 99.19 | 97.01 |
| XXKL13 | 350 | (150:150) | 25021930 | 7.51 | 52 | 98.58 | 95.57 | 24144940 | 96.50 | 99.15 | 96.83 |
| XXKL14 | 350 | (150:150) | 20989599 | 6.30 | 52 | 98.43 | 95.09 | 20222616 | 96.35 | 99.01 | 96.40 |
| XXKL15 | 350 | (150:150) | 22098512 | 6.63 | 52 | 98.25 | 94.60 | 21233740 | 96.09 | 98.94 | 96.10 |
| XXKL16 | 350 | (150:150) | 38002290 | 11.40 | 64 | 92.09 | 79.52 | 18126447 | 47.70 | 99.64 | 98.42 |
| XXKL17 | 350 | (150:150) | 21273358 | 6.38 | 55 | 97.80 | 93.70 | 19522888 | 91.77 | 99.08 | 96.59 |
| XXKL18 | 350 | (150:150) | 24733354 | 7.42 | 58 | 97.13 | 92.30 | 19959635 | 80.70 | 99.64 | 98.45 |
| XXKL19 | 350 | (150:150) | 27885987 | 8.37 | 51 | 98.29 | 94.69 | 26889196 | 96.43 | 98.94 | 96.09 |
| XXKL20 | 350 | (150:150) | 24232970 | 7.27 | 53 | 99.08 | 97.13 | 23553346 | 97.20 | 99.58 | 98.25 |
| XXKL21 | 350 | (150:150) | 29739066 | 8.92 | 56 | 98.44 | 95.70 | 27562667 | 92.68 | 99.58 | 98.26 |

1. InsertSize(bp): InsertSize is the length selected when cutting the gel during library construction. An appropriate InsertSize can prevent contamination of the sequencing connector; 2.SeqStrategy: Sequencing strategy (generally two-ended, each 150bp);
2. RawReads(#): Sequence the number of Raw reads;
3. Raw Base(GB): The number of Raw reads in GB, or the total number of bases in the sequencing Raw data, is calculated by multiplying the number of Raw reads by the sequencing length;
4. %GC: The percentage of G/C bases to the total number of bases;
5. Clean Reads(#): The number of Clean reads obtained after filtering (quality control and de-host sequence);
6. Cleaned(%): The percentage of the remaining sequences after filtering to Raw reads;
7. Q20: The proportion of bases with a mass score higher than 20;
8. Q30: The proportion of bases with a mass score higher than 30.
